# Colorimetric Paper-Based Analytical Devices (PADs) Backed by Chemometrics for Pd(II) Detection

**DOI:** 10.3390/s23177425

**Published:** 2023-08-25

**Authors:** Giancarla Alberti, Lisa Rita Magnaghi, Marzia Iurato, Camilla Zanoni, Raffaela Biesuz

**Affiliations:** 1 Department of Chemistry, University of Pavia, Via Taramelli 12, 27100 Pavia, Italy; lisarita.magnaghi@unipv.it (L.R.M.); rbiesuz@unipv.it (R.B.); 2 Unità di Ricerca di Pavia, Consorzio Interuniversitario Nazionale per la Scienza e Tecnologia dei Materiali (INSTM), Via G. Giusti 9, 50121 Firenze, Italy

**Keywords:** palladium(II), colorimetric Paper-based Analytical Devices (PADs), chemometrics, Partial Least Square regression (PLS), analytical method, colorimetric sensors, metal-ions sensing

## Abstract

This paper presents the development of cheap and selective Paper-based Analytical Devices (PADs) for selective Pd(II) determination from very acidic aqueous solutions. The PADs were obtained by impregnating two cm-side squares of filter paper with an azoic ligand, (2-(tetrazolylazo)-1,8 dihydroxy naphthalene-3,6,-disulphonic acid), termed TazoC. The so-obtained orange TazoC-PADs interact quickly with Pd(II) in aqueous solutions by forming a complex purple-blue-colored already at pH lower than 2. The dye complexes no other metal ions at such an acidic media, making TazoC-PADs highly selective to Pd(II) detection. Besides, at higher pH values, other cations, for example, Cu(II) and Ni(II), can interact with TazoC through the formation of stable and pink-magenta-colored complexes; however, it is possible to quantify Pd(II) in the presence of other cations using a multivariate approach. To this end, UV-vis spectra of the TazoC-PADs after equilibration with the metal ions solutions were registered in the 300–800 nm wavelength range. By applying Partial Least Square regression (PLS), the whole UV-vis spectra of the TazoC-PADs were related to the Pd(II) concentrations both when present alone in solution and also in the presence of Cu(II) and Ni(II). Tailored PLS models obtained with matrix-matched standard solutions correctly predicted Pd(II) concentrations in unknown samples and tap water spiked with the metal cation, making the method promising for quick and economical sensing of Pd(II).

## 1. Introduction

The content of heavy metal ions in the environment, especially in natural waters, has been growing with the increase of human and industry activities, such as the plating and electroplating manufacturing, mining, tanning, textile and rayon industries, batteries, bioreactors, metal smelting, petrochemicals, paper manufacturing, and electrolysis applications [1]. Moreover, the leaching of heavy metal ions from corroded pipelines, unhygienic drinking water practices, soil erosion, sedimentation, resuspension, and evaporation of heavy metal ions from water resources to soil and groundwater are other factors associated with heavy metal ions pollution in the environment [1,2,3]. Heavy metals are non-biodegradable; consequently, they tend to bioaccumulate in living organisms. As they are persistent, they can affect (directly or indirectly) various organisms because of their biomagnification [1,4].

Heavy metals and metalloids are classified as elements with atomic densities higher than 4 g cm^−3^; this class includes nickel, copper, zinc, cadmium, silver, mercury, lead, arsenic, iron, chromium and platinum-group elements [4].

Platinum-group metals, comprising palladium, platinum, rhodium, ruthenium, osmium, and iridium, are naturally found at very low concentrations in the Earth’s crust [5]. They are currently used in many modern technologies and industrial processes because of their peculiar physical-chemical properties [5,6]. For example, these metals are often employed as efficient catalysts to synthesize drug compounds [5,7,8,9]. Platinum-group metals are also an essential part of vehicle catalyst systems to reduce the emission of gaseous pollutants, such as nitrogen oxides, carbon monoxide and hydrocarbons [5,10]. Consequently, due to the increased numbers of cars, the released concentration of Pd, Rh and Pt have risen rapidly in the environment [5,10,11,12,13,14,15].

Other significant sources of palladium-group metals in the environment are wastewater oncological treatment plants, platinum and, recently, some rhodium and palladium complexes are potent cytostatics and are used as chemotherapy drugs [5].

A significant risk of increasing concentrations of the platinum-group metals in the environment is due to insufficient information on their toxicity, effects on human health and the consequence of their bioaccumulation [5]. Only a few studies and the World Health Organization (WHO) have provided little information about the impact of these metals on the environment, and these studies concern only platinum and palladium [12,16,17,18,19,20].

Some toxicological studies have indicated that Pd, being transported to biological systems, accumulates in the food chain, resulting in potential health risks [17,21].

The development of reliable methods for quantifying palladium in environmental and biological matrices is thus a primary focus of attention. 

The most employed instrumental analytical techniques for palladium determination are atomic absorption spectrometry (AAS), inductively coupled plasma–mass spectrometry (ICP-MS), inductively coupled plasma-atomic emission spectrometry (ICP-AES) and neutron activation analysis (NAA) [22]. Most of these methods present high sensitivity but are bulky and expensive. In addition, they require time-consuming sample preparation, sometimes including separation and preconcentration steps before the instrumental analysis [22,23,24].

Few works suggested the application of electrochemical methods that use mercury electrodes for palladium determination. The stripping voltammetry was generally employed, ensuring high sensitivity, low detection limits, and excellent reproducibility. However, these methods are ineffective for routine analyses due to the problems related to mercury’s toxicity and disposal [25,26,27].

Significant advances have been made in developing sensors devoted to Pd(II) detection. Despite a couple of examples where electrochemical sensors were proposed [28,29], in most cases, these are fluorimetric and colorimetric [30,31,32,33].

The development of colorimetric sensors based on solid-material substrates for qualitative and quantitative analyses is one of the most interesting and promising strategies for metal ions sensing. These devices have several advantages: they are sensitive, practical, and adapt to inexperienced users. Among them, Paper-based Analytical Devices (PADs) were widely used for environmental analyses, food controls and clinical trials due to their easy preparation and quick response pros [34,35,36].

The capillary properties of paper provided by the cellulosic fiber network eliminate the necessity of pumping methods instead ofconventional microfluidic devices. In addition, the paper’s ample availability, biodegradability, low price, and ease of surface functionalization make it an attractive substrate in sensors’ development [36,37,38].

Using colored complexes is a helpful strategy for colorimetric sensing on PADs and has been employed for detecting metal ions in different matrixes [39,40,41,42].

The present work fits this scenario; chemometric-assisted colorimetric PADs were developed for Pd(II) detection.

The PADs proposed here were prepared simply with filter paper. In the recent literature, similar devices were realized using different procedures of paper pattering, such as screen-printing, wax printing or laser printing, inkjet etching, photolithography and plasma treatment [43,44,45,46,47,48,49]. These techniques are expensive and sometimes require complicated procedures, making them unaffordable for limited resources laboratories. Moreover, PADs’ wax-patterning needs rigorous flow control of the hydrophobic barrier into the porous matrix of the paper, causing poor homogeneity and, consequently, irreproducibility [46]. An alternative method is a paper cutting into the desired shapes and dimensions, bypassing the need to create the liquid’s containment barriers [37]. This last approach was adopted since the present study aimed to develop low-cost PADs using green materials and simple techniques.

**Figure 1:**
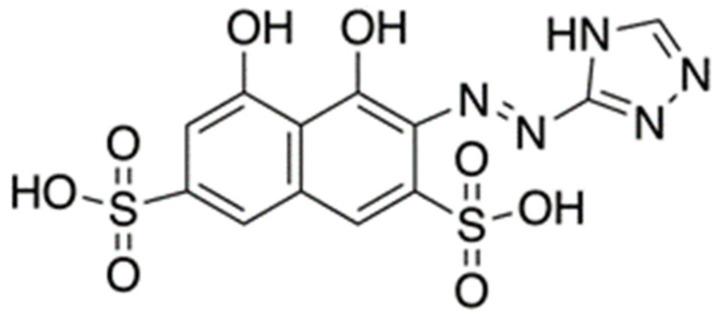
Structure of TazoC.

TazoC (disodium 2-[(1H-5-tetrazolyl)azo]-1,8-dihydroxynaphthalene-3,6 disulphonate, C_11_H_6_O_8_N_6_S_2_Na_3_·3 H_2_O, see the structure in Figure 1), an azo dye, was chosen as the chromophore receptor. This ligand makes the solutions in which it is dissolved and the solid phases impregnated by it orange-red colored. TazoC is not commercially available, but it has already been studied as a complexing ligand for several metal ions [32,50,51,52,53]; in particular, it forms a very stable 1:1 complex with Pd(II), even in strongly acidic solutions. The complex Pd(II)/TazoC is purple-blue, very different from the free ligand’s red-orange: this color change is the propriety used in the development of optical sensors [32]. 

PADs’ ability to detect Pd(II) was first studied at low pH (pH = 2) based on previous knowledge of the complex formation in aqueous solution [51]. The dye does not complex other metal ions at such an acidic media, making TazoC-based PADs highly selective to Pd(II) detection. However, at higher pH values, other cations, such as Cu(II) and Ni(II), can be complexed by TazoC; in this case, the Pd(II) quantification is still possible by applying a multivariate approach. In particular, Partial Least Squares regression (PLS) modeling [54,55,56] was employed here to relate the Pd(II) concentration to the PADs’ visible spectrum. Differently from the univariate approach, the capability of chemometrics tools to extract a vast quantity of information can improve the performances of the PADs to discriminate and classify samples or for multiple-analytes detection [36,57,58,59,60,61]. Moreover, the proper application of chemometric tools to paper-based devices increases their accuracy, reliability, and robustness, thus strengthening their potential [61].

Few other studies have developed paper-based colorimetric sensors for Pd(II). These are mainly test strips obtained by impregnating filter paper with commercially available or newly synthesized fluorescent or colorimetric probes [62,63,64,65,66]. In all these studies, a semi-quantitative analysis was performed based on a color variation, evident to the naked eye, or a simple univariate calibration in standard Pd(II) aqueous solutions. Indeed, the aim was the development of prototypes or test strips with presence/absence response.

With this study, however, we want to demonstrate how an accurate quantitative analysis can be obtained with simple and cheap PADs assisted by proper chemometric tools.

## 2. Materials and Methods

Cellulose filter paper Whatman grade 1 (180 μm thickness, 11 μm-particle retention, 150 s/100 mL speed (Herzberg), and 0.25 psi wet burst, was obtained from Laboindustria S.p.A. (Arzergrande, Padova, Italy). Hydrochloric acid (Suprapur^®^, 36%, Supelco, Darmstadt, Germany,), sodium hydroxide (reagent grade, 97%, powder, Sigma-Aldrich, Saint Louis, MO, USA), acetic acid (glacial, ReagentPlus^®^, ≥99%, Sigma-Aldrich, Saint Louis, MO, USA), and sodium acetate (ACS reagent, ≥99.0%, Sigma-Aldrich, Saint Louis, MO, USA) were obtained from Merk Life Science S.r.l. (Milan, Italy); they were used to prepare the buffer solutions. Palladium standard, *Trace*CERT^®^, 1 g/L Pd in hydrochloric acid, Copper standard, *Trace*CERT^®^, 1 g/L Cu in nitric acid and Nickel standard, *Trace*CERT^®^, 1 g/L Ni in nitric acid (Supelco, Darmstadt, Germany) were obtained from Merk Life Science S.r.l., Milan, Italy and used to prepare diluted standard solutions. 

TazoC was synthesized and purified according to previously described procedures [50]; its empirical formula and molecular weight (536.34 g/mol) were confirmed by elemental analysis. A 1 mM ligand stock solution was prepared by dissolving the weighted solid in ultrapure water.

Tap water samples were obtained from the drinking water supply of Pavia, Italy. Samples were collected from the lab’s sink (Department of Chemistry, University of Pavia, Italy) after flushing cold water for 15 min; they were subsequently acidified to pH 2 with HCl.

**Figure 2:**
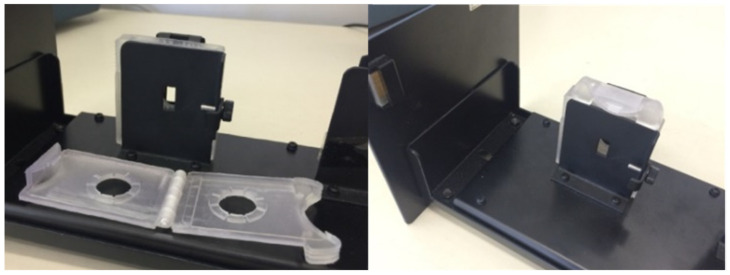
The homemade clip customized to the FLH-740 film holderof the Jasco V-750 UV-vis spectrophotometer.

UV/vis spectra of the PADs were recorded with a Jasco V-750 spectrophotometer equipped with an FLH-740 film holder and a homemade clip designed to make the spectrum acquisition quick and easy (see Figure 2).

The pH of the solutions was checked by a pH meter SevenMulti with an InLab Pro combined glass electrode (Mettler Toledo S.p.A.—Milan, Italy).

**Figure 3:**
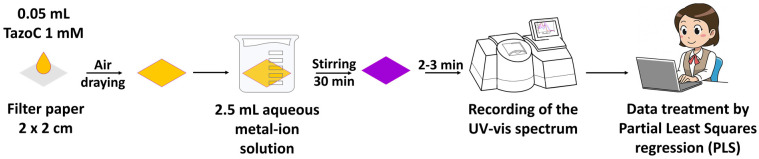
Schematic sketch of the TazoC-PADs preparation, measurement and data treatment.

The cellulose filter paper was cut into 2 cm-sided squares using scissors. Each square, placed on a flat and clean surface, was drop-coated with 0.05 mL of 1 mM TazoC solution and left to air dry. The so-obtained PADs were immersed in 2.5 mL of aqueous metal-ion solution and kept under gentle stirring on a reciprocating shaker for 30 min, i.e., the time required to obtain a homogeneous and stable coloration of the PAD. The PADs were removed from the solution using plastic tweezers and left to air dry for 2–3 min on a flat, clean surface (because the temperature fluctuation affects the PAD’s spectrum, the measurements were performed at 25 °C, setting the air conditioner at that value and with the PADs not completely dry but slightly moistened). Subsequently, the PADs were inserted into the sample holder using plastic tweezers for the UV-vis analysis. The spectrum was registered in the wavelength range from 300 to 800 nm (bandwidth 0.2 nm, scan rate 200 nm/min) against a blank PAD wetted only with the buffer solution. Figure 3 shows a schematic sketch of the TazoC-PADs preparation, measurement, and data treatment.

Partial Least Square regression (PLS) was used for the chemometric data treatment.

The PLS bases have been widely described [54,55,56,67,68,69,70], so they are only brief details here. PLS is a multivariate calibration algorithm aimed to extract most of the information contained in the UV-vis spectra (X variables), which is correlated to the Pd(II) concentration (Y variable) through the so-called latent variables (LVs). Each LV is a linear combination of the spectrum’s absorbances. Unlike PCA, PLS finds directions of the latent variables, explaining the maximum variance and maximizing the correlation with the response. The new coordinates of each object (the UV-vis spectrum) in the space of these latent variables are called scores, whereas the contribution of each spectral variable on the latent variables is called weight or loading.

The PLS model can be written as Y = X·b, allowing the concentration (Y) prediction from the measured spectrum (X). Specifically, X is the *n* × *m* matrix of input data (absorbance values) obtained from the UV-vis spectrum: *n* is the number of samples, and *m* is the number of wavelengths of the whole spectrum. Y is the *n* × *c* concentration matrix, whereas *c* is equal to 1 in PLS1 models (which means when one analyte at the time is determined), and b is the *n* × *c* column vector of regression coefficients, solved when the model is calibrated. Knowing b, the PLS model can predict new Y concentration values from the UV-vis spectra of samples at unknown Pd(II) content. 

The PLS models were built by selecting a suitable data set (training set) obtained with standard solutions at different Pd(II) content to cover the entire experimental domain homogeneously; three PLS models were developed at three different pH values (pH 2, pH 4, and pH 5.5). The training set for each model comprised the data of three replicates of 8-point calibrations (24 rows and 103 columns, i.e., 8 concentrations per 3 replicates and the absorbance values per 103 wavelengths). Each PLS model was tested by a cross-validated procedure on the training set and then on an external test set. Independent Pd(II) solutions were prepared for this purpose; the test set matrix comprised 9 rows (3 concentrations per 3 replicates) and 103 columns (absorbance values per 103 wavelengths).

Moreover, other datasets, comprising mixtures of Pd(II) and Cu(II) at pH 4 and Pd(II), Cu(II) and Ni(II) at pH 5.5 at different metal ion content, were prepared to evaluate the robustness of the method submitted to interferent cations.

A new training set was also prepared to build the PLS model for matrix-matched Pd(II) solutions (tap water). Then, three independent tap water samples were fortified with different Pd(II) concentrations and used as the test set. From the predicted concentrations, the recovery percentages were calculated.

For all PLS models, only the centering pretreatment of data was applied. The chemometric data treatment was performed by the open-source R-based software CAT (Chemometric Agile Tool, version 3.1.2) [71]. 

The tables with the metal ions concentrations for each dataset, the experimental and fitted Pd(II) concentrations and the graph of PLS models’ performances were reported in the Supplementary Materials.

## 3. Results and Discussion

**Figure 4:**
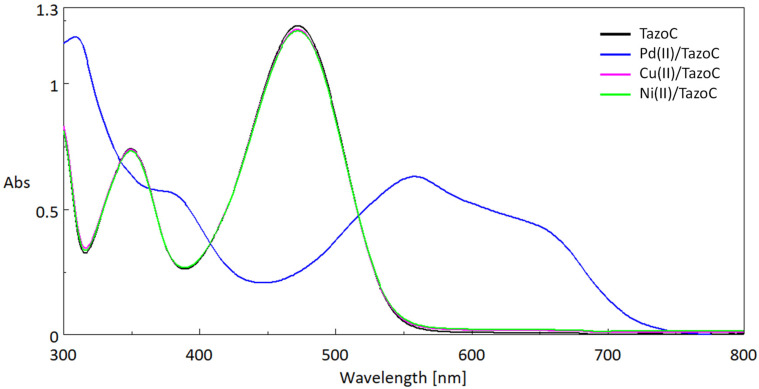
UV-vis spectra (Absorbance (Abs, a.u.) vs. Wavelength (nm)) in aqueous solutions at pH 2 (HCl 0.01 M) of 75 μM Tazo C (black line); 75 μM TazoC and 75 μM Pd(II) (blue line); 75 μM TazoC and 75 μM Cu(II) (pink line); 75 μM TazoC and 75 μM Ni(II) (green line).

The PADs were prepared by cutting a sheet of filter paper into two cm-sided squares. As stated in the introduction, TazoC was chosen as the chromophore receptor since it forms a purple-blue-colored stable 1:1 complex with Pd(II) also in very acidic media; this property has been exploited to develop the present devices. Indeed, until pH 2, only Pd(II) forms a strong complex with TazoC, as evident in Figure 4, where the spectra of the free TazoC and the equimolar aqueous solutions of the ligand with Pd(II), Cu(II) and Ni(II) at pH 2 are reported.

The typical peak of the free ligand at about 475 nm (ε = 17,333 cm^−1^ M^−1^) is in agreement with the data previously reported [51], and the spectrum does not change if other cations than Pd(II) are added at pH 2. As previously observed [51], the absorption maximum of the complex formed with palladium(II) is red-shifted compared to that of the free ligand and well distinguishable; moreover, the peak of the free ligand disappeared, confirming the stoichiometry 1:1 of the complex. In addition, the Pd(II)/TazoC spectrum presents a broad and not well-defined peak, justifying the need for a multivariate calibration tool to quantify Pd(II). 

**Figure 5:**
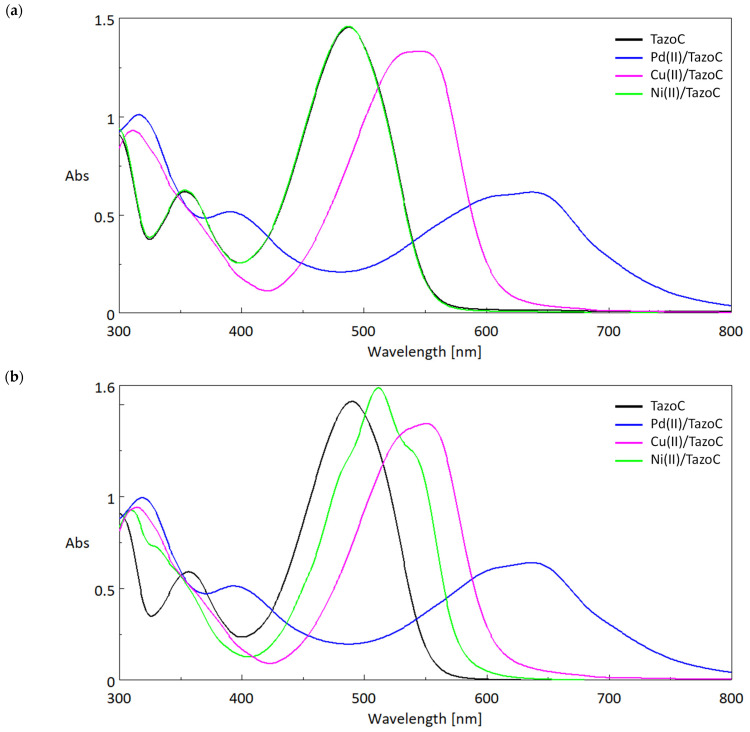
UV-vis spectra (Absorbance (Abs, a.u.) vs. Wavelength (nm)) in aqueous solutions of 75 μM Tazo C (black line); 75 μM TazoC and 75 μM Pd(II) (blue line); 75 μM TazoC and 75 μM Cu(II) (pink line); 75 μM TazoC and 75 μM Ni(II) (green line), (**a**) acetate buffer pH 4, (**b**) acetate buffer pH 5.5.

Figure 5a,b show the spectra obtained in aqueous solution at higher pH values, i.e., pH 4 and 5.5, respectively. These pH values ensure the quantitative formation of the 1:1 TazoC complexes with Cu(II) and Ni(II), as previously verified [53], avoiding the precipitation of the respective hydroxides. 

From the spectra, it is clear that Cu(II) at pH 4 and both Cu(II) and NI(II) at pH 5.5 can cause interference with the Pd(II) determination since their peaks are partially overlapped with that of the target analyte. Even more reason, in this case, to use the multivariate regression PLS is necessary to perform the quantitative analysis, especially for data treatment of the PADs’ spectra.

0.05 mL of 1 mM TazoC was used to load the filter paper since this volume is enough to cover the PAD’s surface entirely without obtaining overflow of the ligand. As an alternative method, the immersion of the PAD in 2.5 mL of 1 mM TazoC solution was tested, but low sorption kinetic and inhomogeneity of the PAD’s color were verified.

It was then necessary to decide the modality to contact the sample solution with the PADs since it could affect the color homogeneity and intensity. Two approaches were tested: drop-coating with 0.5 mL of the solution or immersion of the PAD in 2.5 mL of aqueous metal-ion solution. The better uniformity of the color was achieved with the second strategy, which was thus adopted for all experiments. 

The time required to obtain uniform PAD color and avoid the leaching of the ligand was about 30 min with a gentle stirring on a reciprocating shaker.

Other kinds of filter papers were tested, hoping to reduce the immersion time, but due to the analogous porosity, their use does not confer advantages.

Regarding PADs’ stability, it should be highlighted that these devices are disposable; moreover, the leaching of the chromophore receptor or its complexes with the metal ions can occur if they are stored in aqueous solutions for more than 1 h, so the analysis with the PADs must be immediately performed after their preparation.

**Figure 6:**
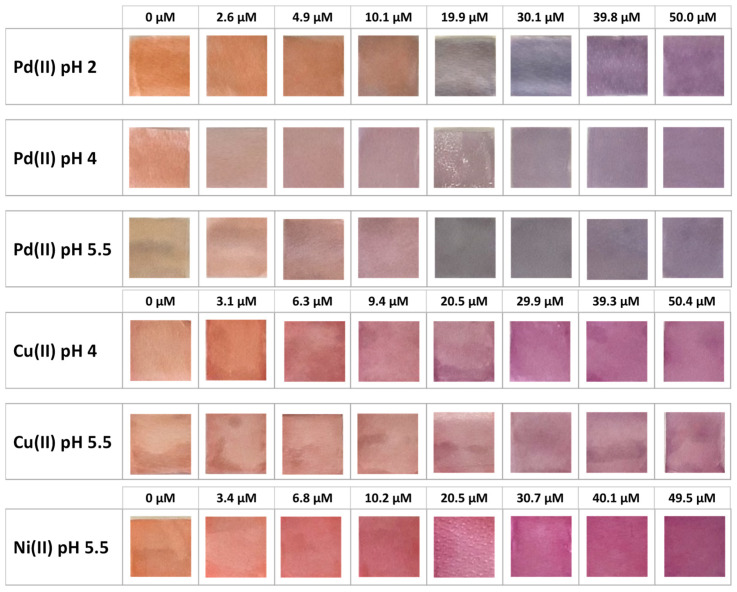
TazoC-PADs after immersion in Pd(II), Cu(II) and Ni(II) solutions at different concentrations and pHs.

**Figure 7:**
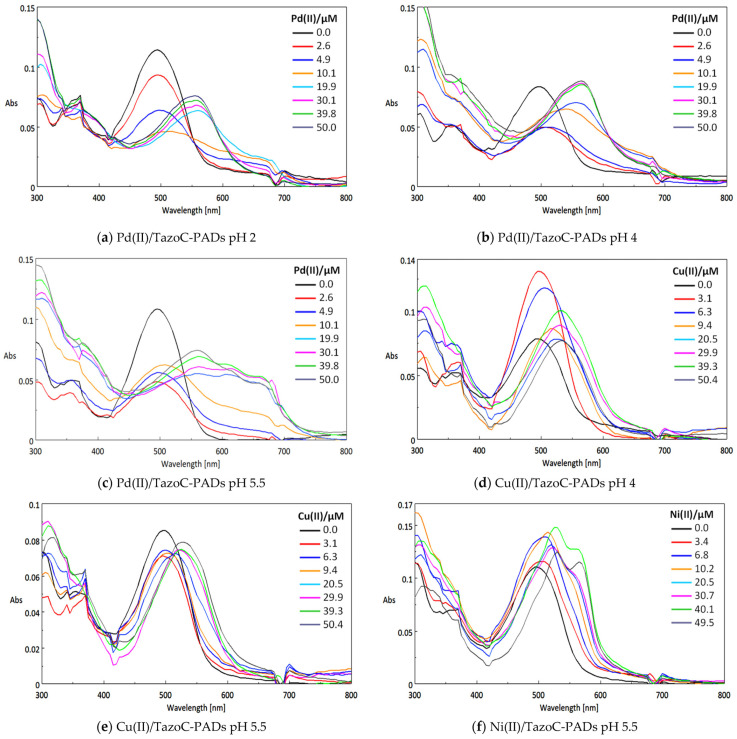
Spectra (Absorbance (Abs, a.u.) vs. Wavelength (nm)) of TazoC-PADs after immersion in Pd(II), Cu(II) and Ni(II) solutions at different concentrations and pHs. The images of the PADs are shown in Figure 5.

Figure 6 shows the TazoC-PADs after contact with the metal ions solutions at different concentrations and pHs, and Figure 7 shows the corresponding UV-vis spectra.

Obviously, PADs spectra are somewhat disturbed compared with those in aqueous solution, but the peak’s shape and position are similar. Therefore, it can be confirmed that the paper does not interfere in the formation of the TazoC complexes with the cations studied. It can also be observed that in all cases, the spectra of the complexes are red-shifted with respect to the free-metal TazoC-PAD spectrum. Clearly, the greater shift is noted for palladium, for which the coloration of the complex is more different from that of the other two cations (see the pictures of the PADs in Figure 6).

As stated above, Partial Least Square regression (PLS) was used for the chemometric data treatment.

The first PLS model relates to the simplest system, i.e., Pd(II)/TazoC-PADs in aqueous solutions at pH 2 (HCl 0.01 M). 

This model was built with a training set comprising the data of three replicates of an 8-point calibration with the Pd(II) concentration ranging from 2.6 to 50 μM. The test set to validate and prove the model’s robustness comprised three replicates of different TazoC-PADs immersed in three Pd(II) solutions (7.5, 25.2, 42.5 μM).

A 5-latent variables model was developed: it ensures 98.61% of explained variance in Cross-Validation (% E.V. in CV), a global Root Mean Square Error in CV (RMSECV) of 2.05 μM and, as regards the test set samples, 1.86 μM of Root Mean Square Error in Prediction (RMSEP). The model performance graph is reported in the Supplementary Material (Figure S1).

**Figure 8:**
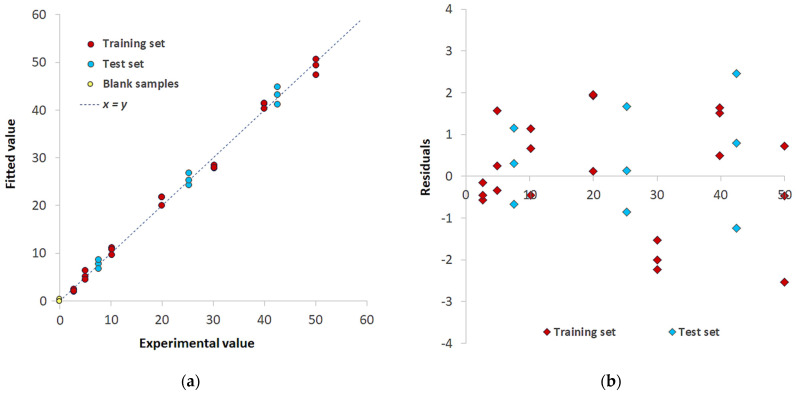
PLS model for Pd(II)/TazoC-PADs at pH 2 (**a**) Experimental values vs. Fitted values plot for the training set (burgundy-colored points) and test set (light blue-colored points) and blank samples (yellow-colored points); (**b**) residuals for the training set (burgundy-colored points) and test set (light blue-colored points).

Figure 8a shows the Experimental vs. Fitted values plot for the training set (burgundy-colored points) and test set (light blue-colored points); samples are similarly distributed alongside the y = x straight line, and no significant difference in the fitting error appears. The residuals plot is reported in Figure 8b; a random distribution around 0 was observed, and a point cloud without a particular structure (the points are placed between the values −3 and 3) justifies the model’s linearity and homoscedasticity assumptions.

Operational values of LOD and LOQ were also obtained by projecting in the PLS model the spectra of 10 blank samples (i.e., TazoC-PADs contacted with solutions at pH 2, without Pd(II), see the yellow-colored points in Figure 7a); the LOD and the LOQ were then computed as 3.3 times and 10 times, respectively, the standard deviation of the predicted blank concentrations. The values are reported in Table 1. This procedure is not precisely rigorous in the case of PLS, but it seems a reasonable estimate since the predicted values are around zero with a very low standard deviation [32]. The authors know that the correct procedure for LOD and LOQ evaluation in PLS analysis is still an open question.

**Figure 9:**
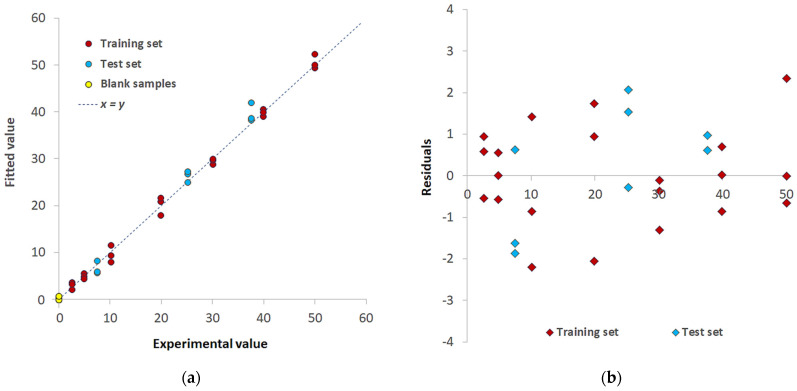

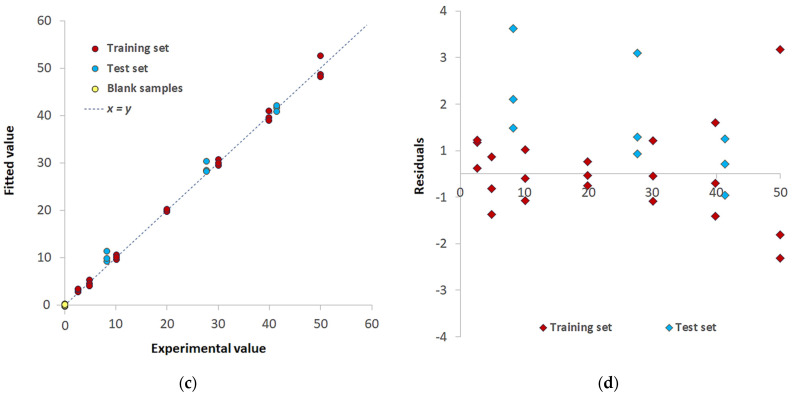
PLS models for Pd(II)/TazoC-PADs at pH 4 and Pd(II)/TazoC-PADs at pH 5.5 (**a**,**c**) Experimental vs. Fitted values plot for the training set (burgundy-colored points), test set (light blue-colored points) and blank samples (yellow-colored points); (**b**,**d**) residuals for the training set (burgundy-colored points) and test set (light blue-colored points). Datasets composition, fitted values and graphs of the models’ performances are reported in the Supplementary Materials.

Figure 9 shows similar plots obtained for the systems Pd(II)/TazoC-PADs at pH 4 and Pd(II)/TazoC-PADs at pH 5.5. 

The figures of merit and the LOD and LOQ values for all three PLS models were summarized in Table 1.

The three models’ robustness and predictive ability were demonstrated; indeed, the training set and test set samples are analogously distributed alongside the y = x straight line, and no significant differences in the fitting errors arise. As expected, the operational LOD and LOQ values are similar independently of the solution pH since the quantitative formation of the Pd(II)/TazoC-PADs complex also occurs in very acidic media.

In addition, the lowest quantifiable concentration values (LOQ) achieved were similar or, in most cases, lower than those obtained with previously proposed test strips based on different colorimetric or fluorescent probes (see Table 2).

Moreover, according to the WHO threshold limit for Pd content in drug chemicals (from 47.0 μM to 94.0 μM, i.e., from 5 mg/L to 10 mg/L) [72], and the United Nations Food and Agriculture Organization (FAO) recommended maximum level for irrigation waters of 47.0 μM (5 mg/L) [73], the lowest quantifiable Pd(II) concentration obtainable with the proposed PADs meet the requirements of WHO and FAO for palladium(II) detection in that matrixes.

**Figure 10:**
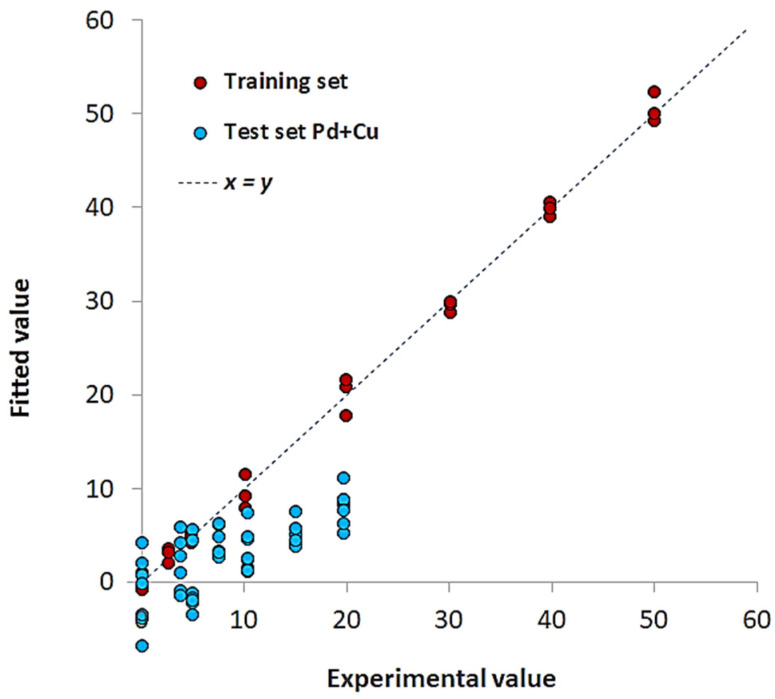
PLS model for Pd(II)/TazoC-PADs at pH 4 Experimental values vs. Fitted values plot for the training set (burgundy-colored points) and Pd(II)/Cu(II) mixtures as test set samples (light blue-colored points).

As for any analytical method subject to interference or in the presence of complex real matrices, calibration with external standards is often ineffective for analyte quantification [74,75,76]. As a demonstrative example, Figure 10 shows the PLS plot of the model Pd(II)/TazoC-PADs at pH 4, where mixtures of Pd(II) and Cu(II) at different concentrations were used as test set samples. The model failed: the Pd(II) concentrations were incorrectly predicted.

The usual strategy to overcome interferences and complex matrix problems is the application of the standard additions method or the matrix-matched calibrations [74,75]. The second approach was adopted here, developing tailored PLS models both in the case of interferents and for spiked tap water samples analysis.

**Figure 11:**
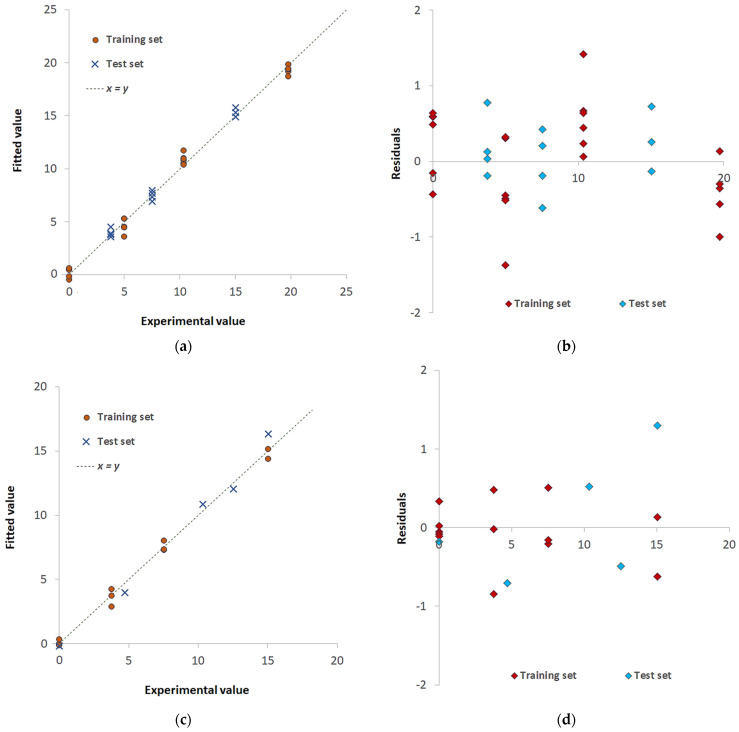
PLS models for Pd(II)+Cu(II)/TazoC-PADs at pH 4 and Pd(II)+Cu(II)+Ni(II)/TazoC-PADs at pH 5.5 (**a**,**c**) Experimental vs. Fitted values plot for the training set (burgundy-colored points) and test set (light blue-colored points); (**b**,**d**) residuals for the training set (burgundy-colored points) and test set (light blue-colored points). Datasets composition, fitted values and graphs of the models’ performances are reported in the Supplementary Materials.

The following Figure 11 and Table 3 report the PLS plots and the figures of merits for the models named Pd(II)+Cu(II)/TazoC-PADs pH 4 and Pd(II)+Cu(II)+Ni(II)/TazoC-PADs pH 5.5. In these cases, mixtures of variable different and independent concentrations of the metal cations were considered for both training and test set samples (see Supplementary Materials).

Both PLS models proved to be adequate in predicting the Pd(II) concentrations even in the presence of interfering cations since there was pretty good agreement between experimental and fitted values. The residual plots’ analysis demonstrates, also in these cases, the linearity and homoscedasticity of the models as the random distribution of the points.

The applicability of the TazoC-PADs to tap water samples was verified by adopting the same approach of matrix-matched calibration used for the interference tests.

As recommended from the preservation of water samples for metals’ analysis rules [77], acidification at pH < 2 must be done to avoid the precipitation of hydroxides. Accordingly, the PLS model was built using tap water samples, acidified at pH 2 and fortified with Pd(II) (model name: Pd(II)/TazoC-PADs TW). Three tap water samples were acidified and spiked with different Pd(II) concentrations (7.5, 25.2 and 44.6 µM) and used as the external dataset (3 replicates for each sample).

**Figure 12:**
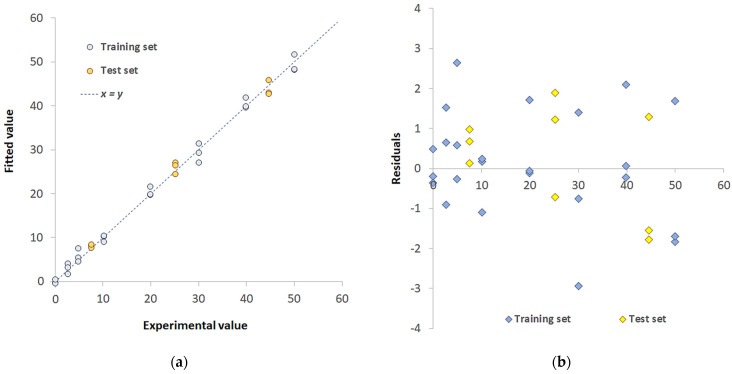
PLS model Pd(II)/TazoC-PADs TW (**a**) Experimental values vs. Fitted values plot for the training set (light blue-colored points) and test set (yellow-colored points); (**b**) residuals for the training set (light blue-colored points) and test set (yellow-colored points). Datasets composition, fitted values and graphs of the model’s performances are reported in the Supplementary Materials.

The PLS model’s figures of merit are reported in Table 4; in Figure 12a, the Experimental vs. predicted values plot for the training set (light blue-colored points) and test set (yellow-colored points) is shown. In Figure 11b, the residual plot is reported. The recovery percentage was computed from the test set samples’ predicted concentration values and reported in Table 5, together with the Pd(II) concentration values obtained by ICP-OES analysis for comparison.

The percentage of recoveries (Rc%) for each Pd(II) concentration is between 80% and 110%, i.e., the acceptable recovery range [78,79], demonstrating the suitability of the proposed method for Pd(II) determination in drinking waters. Moreover, the concentration values obtained are not significantly different from those achieved by the classical ICP-OES technique.

## 4. Conclusions

A cheap, selective colorimetric Paper-based Analytical Device (PADs) for Pd(II) sensing and quantification is proposed. The chromogenic receptor immobilized on filter paper is a non-commercial azo dye named TazoC (disodium 2-[(1H-5-tetrazolyl)azo]-1,8-dihydroxynaphthalene-3,6 disulphonate); it is selected because it forms a strong and stable purple-blue-colored complex with Pd(II) in very acidic media (below pH 2).

A proper application of the chemometric tool, PLS, permitted the building of spectrum/Pd(II) concentration correlation models, taking advantage of using the whole spectrum as the signal accounting for changes in shape and height of the spectrum peaks. Different tailored PLS models were developed and validated, highlighting the need to perform calibrations in the media of interest to overcome interferences and complex matrix problems. The developed PLS models proved to be adequate in predicting the Pd(II) concentrations even in the presence of interfering cations and in real matrices since there was pretty good agreement between experimental and fitted values.

The lowest quantifiable concentration values achieved, of about 2.5 μM, were similar to or, in most cases, lower than those obtained with previously proposed test strips based on different colorimetric or fluorescent probes. In addition, the lowest quantifiable Pd(II) concentration obtainable with the proposed PADs meets the requirements of WHO and FAO for palladium(II) detection in drug chemicals and irrigation waters.

Unlike previously developed paper-based sensors, whose aim was to create test strips with presence/absence response, this study demonstrated how an accurate quantitative Pd(II) analysis should be possible with simple and cheap PADs assisted by chemometric tools.

## Figures and Tables

**Table 1:** Number of latent variables (LVs), % explained variance in cross-validation (%Exp. Var. CV), Root Mean Square Error in CV (RMSECV), Root Mean Square Error in prediction (RMSEP), the correlation coefficient of the regression (r^2^), the limit of detection (LOD) and the limit of quantification (LOQ) for the three PLS models of Figure 8.

		Pd(II)/TazoC-PADs pH 2	Pd(II)/TazoC-PADs pH 4	Pd(II)/TazoC-PADs pH 5.5
Training set	LVs	5	8	8
	%Exp. Var. CV	98.61	96.92	98.02
	RMSECV (μM)	2.05	3.08	2.45
	r^2^ model	0.994	0.996	0.997
Test set	RMSEP (μM)	1.86	1.92	1.55
	r^2^ prediction	0.994	0.991	0.995
Blank samples	LOD (μM)	0.8	0.8	0.7
	LOQ (μM)	2.3	2.4	2.0

**Table 2:** Comparison of the LOQ values obtained with different test strips for Pd(II) sensing.

Sensor	LOQ (μM)	Reference
Rhodamine B-based test papers	2.5	[62]
Coumarin-based test papers	10	[63]
2-(2′-hydroxyphenyl)benzothiazole-based test papers	4.7	[64]
PTAID-based test papers ^1^	100	[65]
SAS-IMIs-based test silica plates ^2^	10	[66]
TazoC-based test papers ^3^	2–2.4	This work

^1^ Purine derivative-based fluorescent probe (PTAID)-based test papers. ^2^ Salicylaldehyde bis-Schiff-base probe (SAS) decorated with imidazolium ionic liquid moieties (IMIs) at both ends adsorbed onto silica-gel-based thin layer chromatography (TLC) plates. ^3^ (2-(tetrazolylazo)-1,8 dihydroxy naphthalene-3,6,-disulphonic acid (TazoC)-based test papers.

**Table 3:** Number of latent variables (LVs), % explained variance in cross-validation (%Exp. Var. CV), Root Mean Square Error in CV (RMSECV), Root Mean Square Error in prediction (RMSEP), and the correlation coefficient of the regression (r^2^), for the PLS models of Figure 10.

		Pd(II)+Cu(II)/TazoC-PADs pH 4	Pd(II)+Cu(II)+Ni(II)/TazoC-PADs pH 5.5
Training set	LVs	8	7
	%Exp. Var. CV	93.07	74.2
	RMSECV (μM)	1.92	2.33
	r^2^ model	0.992	0.995
Test set	RMSEP (μM)	1.41	0.86
	r^2^ prediction	0.993	0.990

**Table 4:** Number of latent variables (LVs), % explained variance in cross-validation (%Exp. Var. CV), Root Mean Square Error in CV (RMSECV), Root Mean Square Error in prediction (RMSEP), and the correlation coefficient of the regression (r^2^), for the PLS model Pd(II)/TazoC-PADs TW.

		Pd(II)/TazoC-PADs TW
Training set	LVs	5
	%Exp. Var. CV	98.73
	RMSECV (μM)	1.96
	r^2^ model	0.995
Test set	RMSEP (μM)	2.06
	r^2^ prediction	0.992

**Table 5:** Recovery test. The number in parenthesis is the standard deviation (*n* = 3).

Pd(II) Added (μM)	Pd(II) Found_ICP-OES_ (µM)	Pd(II) Found_TazoC-PADs_ (µM)	Rc%	E%
7.5	7.4(3)	8.1(5)	108	8
25.2	25.0(4)	26(1)	103	3
44.6	45.2(7)	44(2)	98	−2

## Data Availability

Data is contained within the article and Supplementary Material.

## Supplementary Materials

The following supporting information can be downloaded at: https://www.mdpi.com/article/10.3390/s23177425/s1, Figure S1. PLS model Pd(II)/TazoC-PADs pH 2: Model performances; Table S1. PLS model Pd(II)/TazoC-PADs pH 2: Experimental and fitted data; Figure S2. PLS model Pd(II)/TazoC-PADs pH 4: Model performances; Table S2. PLS model Pd(II)/TazoC-PADs pH 4: Experimental and fitted data; Figure S3. PLS model Pd(II)/TazoC-PADs pH 5.5: Model performances; Table S3. PLS model Pd(II)/TazoC-PADs pH 5.5: Experimental and fitted data; Figure S4. PLS model Pd(II)+Cu(II)/TazoC-PADs pH 4: Model performances; Table S4. Pd(II)+Cu(II)/TazoC-PADs pH 4: Experimental and fitted data; Figure S5. PLS model Pd(II)+Cu(II)+Ni(II)/TazoC-PADs pH 5.5: Model performances; Table S5. Pd(II)+Cu(II)+Ni(II)/TazoC-PADs pH 5.5: Experimental and fitted data; Figure S6. PLS model Pd(II)/TazoC-PADs TW: Model performances; Table S6. PLS model Pd(II)/TazoC-PADs TW: Experimental and fitted data.
