# Supplementary material for: Colorimetric Paper-Based Analytical Devices (PADs) Backed by Chemometrics for Pd(II) Detection
Source: Sensors (Basel). 2023 Aug 25;23(17):7425. doi: 10.3390/s23177425 (PMC10490827; doi:10.3390/s23177425)
Supplement: Supplementary file 1 [file sensors-23-07425-s001.zip › sensors-2567545-supplementary.pdf]

Supplementary Materials

Colorimetric PADs backed by chemometrics for Pd(II) detection

Giancarla Alberti, Lisa Rita Magnaghi, Marzia Iurato, Camilla Zanoni and Raffaela Biesuz

Figure S1. PLS model Pd(II)/TazoC-PADs pH 2: Model performances

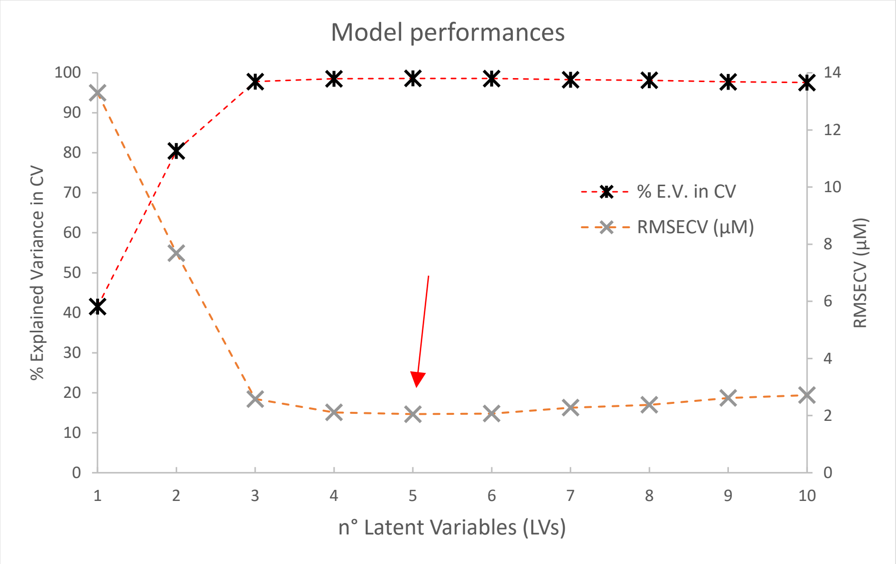

Table S1. PLS model Pd(II)/TazoC-PADs pH 2: Experimental and fitted data

**Training set**		**Test set**	
Exp. [Pd] µM	**Fit.** [Pd] µM	Exp. [Pd] µM	**Fit.** [Pd] µM
2.6	2.1	7.5	7.8
2.6	2.5	7.5	8.7
2.6	2.2	7.5	6.8
4.9	5.1	25.2	24.3
4.9	6.5	25.2	26.8
4.9	4.5	25.2	25.3
10.1	11.3	42.5	43.3
10.1	10.8	42.5	41.2
10.1	9.7	42.5	44.9
19.9	21.9		
19.9	21.9		
19.9	20.0		
30.1	28.5		
30.1	27.8		
30.1	28.1		
39.8	41.4		
39.8	41.5		
39.8	40.3		
50.0	50.7		
50.0	47.4		
50.0	49.5		

Figure S2. PLS model Pd(II)/TazoC-PADs pH 4: Model performances

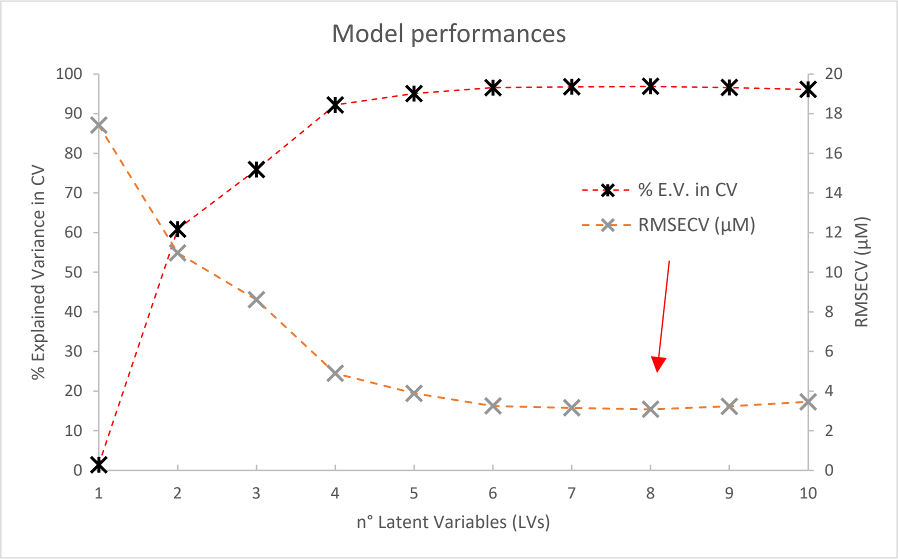

Table S2. PLS model Pd(II)/TazoC-PADs pH 4: Experimental and fitted data

**Training set**		**Test set**	
Exp. [Pd] µM	**Fit.** [Pd] µM	Exp. [Pd] µM	**Fit.** [Pd] µM
2.6	2.1	7.5	5.6
2.6	3.6	7.5	5.9
2.6	3.2	7.5	8.1
4.9	5.4	25.2	24.9
4.9	4.9	25.2	26.7
4.9	4.3	25.2	27.2
10.1	7.9	37.6	38.2
10.1	9.3	37.6	38.6
10.1	11.6	37.6	41.9
19.9	17.9		
19.9	20.9		
19.9	21.7		
30.1	28.8		
30.1	29.7		
30.1	30.0		
39.8	39.0		
39.8	40.5		
39.8	39.9		
50.0	52.3		
50.0	49.3		
50.0	50.0		

Figure S3. PLS model Pd(II)/TazoC-PADs pH 5.5: Model performances

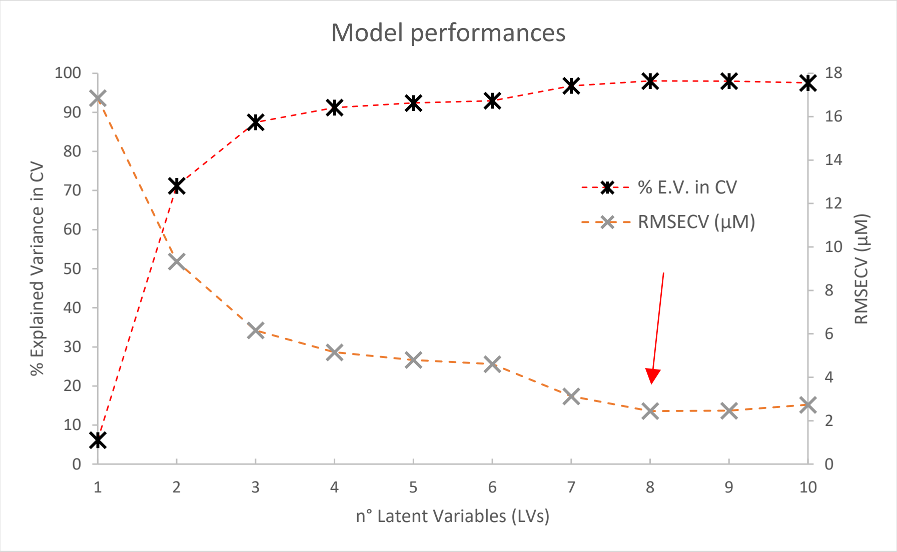

Table S3. PLS model Pd(II)/TazoC-PADs pH 5.5: Experimental and fitted data

**Training set**		**Test set**	
Exp. [Pd] µM	**Fit.** [Pd] µM	Exp. [Pd] µM	**Fit.** [Pd] µM
2.6	2.8	8.3	11.4
2.6	3.3	8.3	9.3
2.6	3.4	8.3	9.9
4.9	5.3	27.7	28.5
4.9	4.6	27.7	28.1
4.9	4.0	27.7	30.3
10.1	10.7	41.3	41.6
10.1	9.6	41.3	42.1
10.1	10.0	41.3	40.9
19.9	19.9		
19.9	19.7		
19.9	20.2		
30.1	29.5		
30.1	30.8		
30.1	30.0		
39.8	39.6		
39.8	40.9		
39.8	38.9		
50.0	48.7		
50.0	48.2		
50.0	52.7		

Figure S4. PLS model Pd(II)+Cu(II)/TazoC-PADs pH 4: Model performances

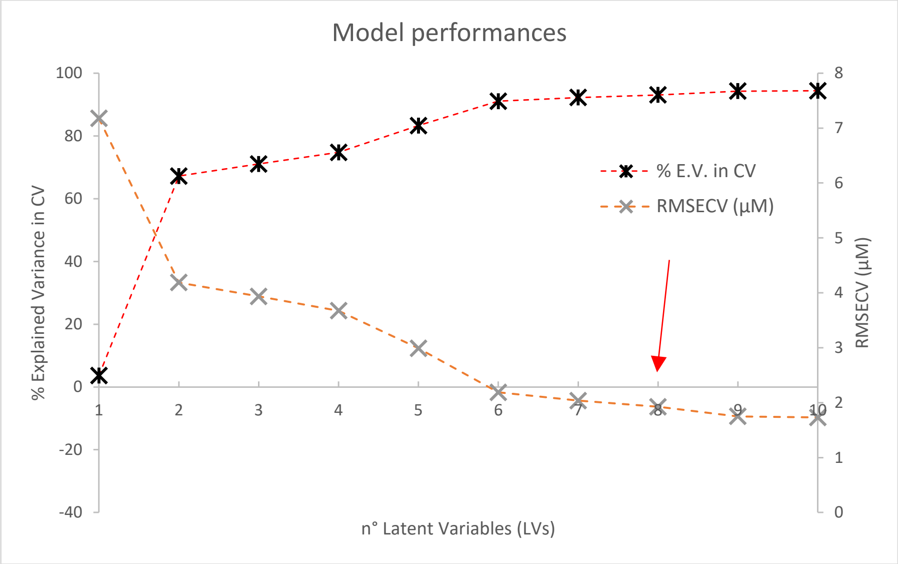

Table S4. PLS model Pd(II)+Cu(II)/TazoC-PADs pH 4: Experimental and fitted data

**Training set**			**Test set**		
**[Cu]** µM	Exp. [Pd] µM	**Fit.** [Pd] µM	**[Cu]** µM	Exp. [Pd] µM	**Fit.** [Pd] µM
5.0	0.0	0.6	20.5	3.8	3.9
10.1	0.0	0.6	0.0	3.8	3.8
20.5	0.0	0.5	20.5	7.5	7.3
0.0	5.0	3.6	0.0	7.5	7.7
5.0	5.0	4.5	5.0	15.0	15.8
10.1	5.0	4.5	10.1	15.0	14.9
0.0	10.3	10.6	3.8	3.8	3.6
10.1	10.3	11.8	14.9	3.8	4.5
5.0	19.7	19.2	3.8	7.5	7.9
10.1	19.7	19.4	14.9	7.5	6.9
20.5	19.7	19.9	3.8	15.0	15.3
3.8	0.0	-0.4			
14.9	0.0	-0.2			
3.8	0.0	0.6			
7.6	5.0	4.5			
14.9	5.0	5.3			
7.6	5.0	5.3			
14.9	10.3	11.0			
0.0	10.3	10.8			
5.0	10.3	11.0			
10.1	10.3	10.4			
20.5	19.7	19.4			
5.0	19.7	18.7			

Figure S5. PLS model Pd(II)+Cu(II)+Ni(II)/TazoC-PADs pH 5.5: Model performances

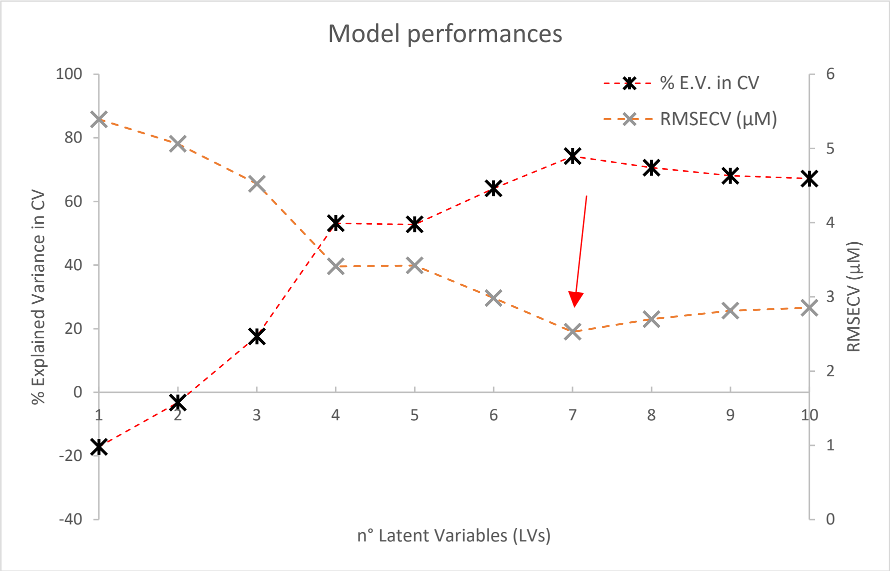

Table S5. PLS model Pd(II)+Cu(II)+Ni(II)/TazoC-PADs pH 5.5: Experimental and fitted data

Training set				Test set			
**[Cu]** µM	**[Ni]** µM	Exp. [Pd] µM	**Fit.** [Pd] µM	**[Cu]** µM	**[Ni]** µM	Exp. [Pd] µM	**Fit.** [Pd] µM
0.0	20.5	0.0	-0.1	4.7	30.7	0.0	-0.2
0.0	30.7	0.0	-0.1	0.0	30.7	4.7	4.0
0.0	40.1	0.0	0.0	15.7	3.7	12.5	12.0
7.9	30.7	0.0	0.3	0.0	30.7	10.3	10.9
9.4	30.7	0.0	-0.1	15.7	0.0	15.0	16.3
3.1	30.7	3.8	3.7				
7.9	15.7	3.8	2.9				
15.7	0.0	3.8	4.2				
0.0	30.7	7.5	7.3				
3.1	0.0	7.5	7.4				
7.9	20.5	7.5	8.0				
3.1	7.5	15.0	14.4				
7.9	0.0	15.0	15.2				

Figure S6. PLS model Pd(II)/TazoC-PADs TW: Model performances

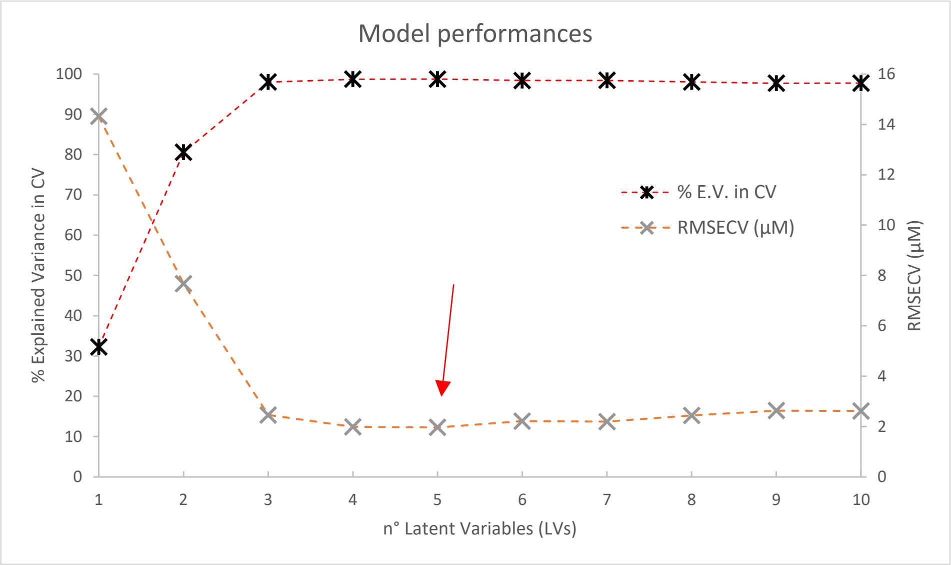

Table S6. PLS model Pd(II)/TazoC-PADs TW: Experimental and fitted data

**Training set**		**Test set**	
Exp. [Pd] µM	**Fit.** [Pd] µM	Exp. [Pd] µM	**Fit.** [Pd] µM
2.6	1.7	7.5	8.2
2.6	4.1	7.5	7.6
2.6	3.3	7.5	8.5
4.9	7.5	25.2	27.1
4.9	5.5	25.2	24.5
4.9	4.6	25.2	26.4
10.1	9.1	44.6	45.9
10.1	10.3	44.6	43.0
10.1	10.4	44.6	42.8
19.9	19.8		
19.9	21.6		
19.9	19.9		
30.1	27.1		
30.1	29.3		
30.1	31.5		
39.8	39.6		
39.8	39.9		
39.8	41.9		
50.0	51.7		
50.0	48.2		
50.0	48.3		
